# Comprehensive validation of halcyon 2.0 plans and the implementation of patient specific QA with multiple detector platforms

**DOI:** 10.1002/acm2.12881

**Published:** 2020-05-05

**Authors:** Eric Laugeman, Ana Heermann, Jessica Hilliard, Michael Watts, Marshia Roberson, Robert Morris, Sreekrishna Goddu, Abhishek Sethi, Imran Zoberi, Hyun Kim, Sasa Mutic, Geoffrey Hugo, Bin Cai

**Affiliations:** ^1^ Department of Radiation Oncology Washington University St. Louis MO USA

**Keywords:** double‐stack MLC, patient‐specific QA, ring gantry LINAC

## Abstract

**Purpose:**

To perform a comprehensive validation of plans generated on a preconfigured Halcyon 2.0 with preloaded beam model, including evaluations of new features and implementing the patient specific quality assurance (PSQA) process with multiple detectors.

**Methods:**

A total of 56 plans were generated in Eclipse V15.6 (Varian Medical System) with a preconfigured Halcyon treatment machine. Ten plans were developed via the AAPM TG‐119 test suite with both IMRT and VMAT techniques. 34 clinically treated plans using C‐arm LINAC from 24 patients were replanned on Halcyon using IMRT or VMAT techniques for a variety of sites including: brain, head and neck, lung, breast, abdomen, and pelvis. Six of those plans were breast VMAT plans utilizing the extended treatment field technique available with Halcyon 2.0. The dynamically flattened beam (DFB), another new feature on Halcyon 2.0, was also used for an AP/PA spine and four field box pelvis, as well as ten 3D breast plans. All 56 plans were measured with an ion chamber (IC), film, portal dosimetry (PD), ArcCHECK, and Delta4. Tolerance and action limits were calculated and compared to the recommendations of TG‐218.

**Results:**

TG‐119 IC and film confidence limits met those set by the task group, except for IMRT target point dose. Forty‐four of 46 clinical plans were within 3% for IC measurements. Average gamma passing rates with 3% dose difference and 2mm distance‐to‐agreement for IMRT/VMAT plans were: Film – 96.8%, PD – 99.9%, ArcCHECK – 99.1%, and Delta4 – 99.2%. Calculated action limits were: Film – 86.3%, PD – 98.4%, ArcCHECK – 96.1%, and Delta4 – 95.7%. Extended treatment field technique was fully validated and 3D plans with DFB had similar results to IMRT/VMAT plans.

**Conclusion:**

Halcyon plan deliveries were verified with multiple measurement devices. New features of Halcyon 2.0 were also validated. Traditional PSQA techniques and process specific tolerance and action limits were successfully implemented.

## 1 Introduction

The deficit of radiotherapy access for low‐ and middle‐income countries has been well‐documented.^1^, ^2^, ^3^, ^4^, ^5^ With expected increasing worldwide cancer incidences, demand for accessible and high quality radiotherapy will intensify.^6^ To address the global need of image‐guided radiation therapy (IGRT), a new linear accelerator (LINAC), Halcyon (Varian Medical System, Palo Alto, CA), was recently released. The Halcyon is a ring‐shaped LINAC with a compact gantry equipped with double stack multileaf collimator (DSMLC) that can provide a single 6MV flattening‐filter‐free (6FFF) beam. It is designed to have short installation and commissioning time with a preconfigured machine and beam model in the treatment planning system (TPS). Additionally, IGRT treatment delivery times can be lessened compared to traditional C‐arm LINACs due to the faster gantry rotation up to 4 rpm.^7^ This allows for low resource clinics with high demand to deliver high quality treatments without sacrificing patient volume.

Due to the preconfigured nature of the beam model, the conventional commissioning concept which uses on‐site collected data to model the beam in the TPS is not applicable. Instead, a comprehensive validation process that ensures the measured results match the TPS is critical before releasing the machine for clinical treatment.^8^, ^9^ Furthermore, with the standardized beam model, a consistency across all clinics and Halcyon units is feasible. Therefore, a more direct comparison to other clinics’ processes can be utilized to ensure that a clinic is meeting minimum standards across the world. One such process that often proves difficult to compare from clinic to clinic is patient‐specific quality assurance (PSQA). With a standard beam model, benchmarking PSQA results with other clinics utilizing the same measurement device becomes feasible, and can provide a resource for comparing and ensuring that a clinic is within universal tolerance limits.

Previous work by De Roover et al,^10^ examined a comprehensive validation of the preconfigured beam model and IMRT/VMAT plan delivery on Halcyon 1.0 system. Several new features included in the 2.0 upgrade related to plan delivery require additional validation. Firstly, the second‐generation double stack MLC (SX2) allows both levels to modulate the beam compared to the first‐generation (SX1) where the proximal leaves only served as a back‐up to the field shaping distal leaves. To increase effective field size, an extended treatment field technique allows for planning with two isocentres at most 8 cm apart in the longitudinal direction that is automatically applied during treatment. Thus, the maximum effective field length increases from 28 cm to 36 cm. A fixed MLC sequence is also available that flattens the FFF beam such that static open field plans may be delivered that are commensurate with the conventional 3D conformal plans.

The goal of this paper is to (a) perform a comprehensive validation of plans generated on a preconfigured Halcyon 2.0 with independently movable DSMLC SX2; (b) evaluate the new features including dynamically flattened beam (DFB) and extended treatment field technique; and (c) implement the PSQA process with multiple detectors and provide a source for other Halcyon users to refer to when commissioning their patient specific quality assurance program. This study incorporates recommendations from AAPM TG‐218^11^ to evaluate agreement with universal limits while performing PSQA on the Halcyon treatment unit.

## 2 Methods and Materials

### 2.A Halcyon 2.0

All measurements were done using the Halcyon 2.0. The Halcyon is a ring‐shaped LINAC with a single 6FFF photon energy and a rotating, double stack MLC, SX2. All MLCs have a width of 1 cm when projected at isocentre (SAD = 100 cm), and the stacks are offset by 0.5 cm. The SX2 collimator allows both stacks of MLCs to independently modulate the beam, providing greater ability to modulate the beam compared to SX1, where the proximal layer (MLC stack closer to the target) only acted as backup to the modulating distal layer (MLC stack closer to the patient). The maximum field size is 28 cm × 28 cm. However, the Halcyon 2.0 enables extended treatment field planning with two isocentres a maximum of 8 cm apart in the longitudinal direction, for a maximum effective field size of 36 cm × 28 cm. The delivery is after a single imaging session, however, the imaging isocentre can be placed at either isocentre or anywhere in between the two. Halcyon 2.0 contains a fixed MLC sequence that flattens the 6FFF beam by sweeping the proximal MLCs while the beam is delivering dose to provide a flattened beam profile (dynamically flattened beam, DFB).^12^, ^13^, ^14^ The distal MLCs define the treatment field.

### 2.B Treatment plans

A total of 56 plans were generated using Eclipse 15.6 (Varian Medical System) with a preloaded 6FFF Halcyon beam model (Anisotropic Analytical Algorithm 15.6.02). Ten plans were developed via the AAPM TG‐119^15^ test suite with both IMRT and VMAT techniques. Clinically treated plans of 24 patients were retrospectively replanned (34 plans) on Halcyon using sliding window IMRT and VMAT for a variety of sites, including brain, head and neck, lung, breast, abdomen, and pelvis. All plans were deemed clinically acceptable. The Halcyon IMRT plans used the same beam arrangement as used in clinical plans. Halcyon VMAT planning started with two arcs and the number of arcs was increased to meet clinical constraints if needed. Michiels et al.^7^ showed a decrease in OAR doses when the number of arcs was increased to three. Six plans were breast VMAT plans utilizing the extended treatment field feature. One AP/PA spine plan, one four field box, and ten 3D breast plans using the DFB were also created and measured. Table 1 shows an overview of all 56 plans. Mean MU ratio (total MU/prescribed dose) for IMRT/VMAT plans is 4.5 [range: 2.0–9.0]. For plans with DFB mean MU ratio is 2.9 [2.5–3.4].

**Table 1:** The number of plans per site and per treatment technique.

Site	# IMRT	# VMAT	3D with DFB
Whole Pelvis	1	3	1
Prostate	2	2	–
Brain	2	2	–
Head and Neck	2	2	–
Lung	2	4	–
Abdomen	1	–	–
Breast (single isocentre)	–	5	–
Breast (extended treatment field)	–	6	–
Breast (3D)	–	–	10
Spine	–	–	1
TG 119	5	5	–

DFB, dynamically flattened beam.

### 2.C Point dose ionization chamber measurements

To validate the IMRT/VMAT field, a micro or scanning chamber is often recommended for absolute dose measurement.^16^ In our study, absolute point dose measurements were made with an ADCL calibrated PTW 31010, 0.125 cc ion chamber (IC). A 15 × 15 × 15 cm^3^ cube solid water phantom with multiple water‐equivalent plastic blocks and spacers was used to place the IC in a high dose, low gradient region of the calculated dose distribution for the clinical plans.^17^ TG 119 IC measurements were done at the prescribed locations.^15^ Measured point doses were compared to point doses calculated at the same location within the phantom and percent differences are reported.

### 2.D Field‐by‐field dose map validation

Portal dosimetry (PD) was used for 2D dose map validation. Halcyon 2.0 is equipped with an aS1200 electronic portal imaging device (EPID). The panel is 43 cm × 43 cm with 1280 × 1280 pixel matrix which makes the spatial resolution 2.98 mm^−1^. The frame refresh rate is 24 frames/s which does not saturate when measuring the nominal 6 FFF beam. The imager is fixed at a source to imager distance (SID) of 154 cm. An Anisotropic Analytical Algorithm (Eclipse, v15.6) was preconfigured for dosimetry calculation. The same algorithm was used in portal dosimetry calculation to predict the fluence pattern. The portal dosimetry model is preconfigured with standard beam data on Halcyon. The measurements were performed at the planned gantry and collimator angles. A composite dose image of all treatment fields was also created. Gamma analysis was performed for each field as well as the composite image with the following parameters based on TG‐218 recommendations: 3% dose difference threshold, 2 mm distance‐to‐agreement (DTA) threshold, global normalization, and 10% lower dose threshold.

### 2.E True composite measurements

True composite (TC) measurements utilize a 3D phantom with film or a detector array inside to sample the fully delivered 3D dose distribution using the actual treatment plan parameters. Absolute TC measurements were done with Delta4 + Phantom (ScandiDos, Sweden) with D4 software (December 2017 release) and ArcCHECK (SunNuclear, Florida, USA) with SNC Patient (v8.0). Relative TC measurements were done with EDR2 radiographic Film (Carestream, New York, USA) at the center of a 30 cm^2^ solid water stack 16 cm in height in the coronal slice with the long edge parallel to the axial direction. The film calibration curve was obtained by irradiating a single film with a nonuniform pattern via a dynamic MLC.^18^ All films were processed, scanned (scan resolution 96 dpi), and analyzed with a commercial film dosimetry system (Radiological Imaging Technology, INC., Denver, Colorado) after obtaining a calibration curve. Following TG‐218 recommendations the following gamma criteria was used: 3% dose difference threshold, 2 mm DTA threshold, global normalization and 10% lower dose threshold.

### 2.F Tolerance and action limits

Utilizing statistical process control method presented by TG‐218^11^ and several groups,^19^, ^20^, ^21^, ^22^ IMRT QA tolerance and action limits were calculated for each measurement device and Halcyon combination. A comparison was done to the recommended universal limits. Tolerance limits were calculated from the lower control limit of an I‐chart,^23^ which also helps to determine if IMRT QA measurements display out‐of‐control behavior over time (Equations 4‐6 in TG‐218^11^). Action limits were calculated using the following equation^24^ (equation 3 in TG‐218^11^):

$$\Delta A=\beta \sqrt{\sigma^{2}+{\left(x-T\right)}^{2},}$$

where x and $\sigma^{2}$ are the process mean and variance, T is the target value (100% for gamma passing rate and 0% for IC measurements), β is a constant assumed to be 6 (based on TG‐218), and ΔA is the difference between the upper and lower action limits. The task group recommended the use of 3% dose difference and 2mm distance‐to‐agreement criteria with 10% dose threshold and global normalization. The suggested universal tolerance and action limits are 95% and 90% points passing respectively.

### 2.G Plan delivery stability

To test the plan delivery stability over time, two plans (IMRT whole pelvis and breast VMAT) were measured three times with the ArcCHECK over 4–6 months and twice with portal dosimetry. Analysis was done consistent with the earlier descriptions. The purpose of this test is to validate the ability of the system to deliver IMRT and VMAT plans consistently over a long period of time.

## 3 Results

### 3.A TG 119 Plans

Table 2 shows the TG‐119 point dose and film results along with calculated confidence levels (|mean| + 1.96σ). These confidence limits quantify the degree of agreement that should be expected between measured and calculated doses. All but two point dose measurements were within 3%. Confidence limits met the TG‐119^15^ recommended limits (4.5% for target structures and 4.7% for avoidance structures) except for the IMRT target confidence limit of 6.4%. This was due to the C‐shape hard constraint plan where the prescribed measurement point landed on a steep gradient within the high dose region. The harder constraints were met with the IMRT plan, but significant dose inhomogeneity resulted within the PTV. A nearby point in a more homogeneous region was measured with percent difference of less than 1%. All film results were above 90% with 3%/2mm gamma criteria, and only one film was below 95% (C‐shape target IMRT with hard constraints). The confidence limits were below 3% for IMRT avoidance structures and both target and avoidance structures for VMAT. IMRT target confidence level was 6.3%, still well within TG‐119 tolerance (12.4%) with a tighter gamma criteria. TG‐119 plan QA results with QA devices other than IC and film are included with the clinical plans as they were delivered and analyzed the same as the clinical plans.

**Table 2:** TG‐119 results for point dose (% difference calculated and measured) and radiographic film measurements (gamma analysis with 3%/2 mm gamma criteria).

Test	location	Point dose (%)		Film (3%/2 mm)	
		IMRT	VMAT	IMRT	VMAT
MultiTarget	Isocentre	−0.6	−0.2	99.6	99.4
	4cm superior	−1.4	−0.8		
	4cm inferior	−0.5	−2.0		
Prostate	Target	−0.4	1.6	99.6	99.7
	OAR	1.1	3.4	98.8	98.9
Head/Neck	Target	−2.5	2.3	96.9	99.0
	OAR	−0.8	−1.6	98.5	99.6
CShape (easy)	Target	−1.8	−0.6	99.8	99.9
	OAR	1.0	1.7	99.9	98.6
CShape (hard)	Target	5.6	2.9	94.7	100.0
	OAR	1.6	−0.1	98.5	99.4
Confidence Limit	Target	6.4	4.1	6.3	1.2
	OAR	2.5	4.2	2.4	2.0
	Combined	4.4	4.2	4.9	1.6

### 3.B Clinical plans

**Fig. 1:**
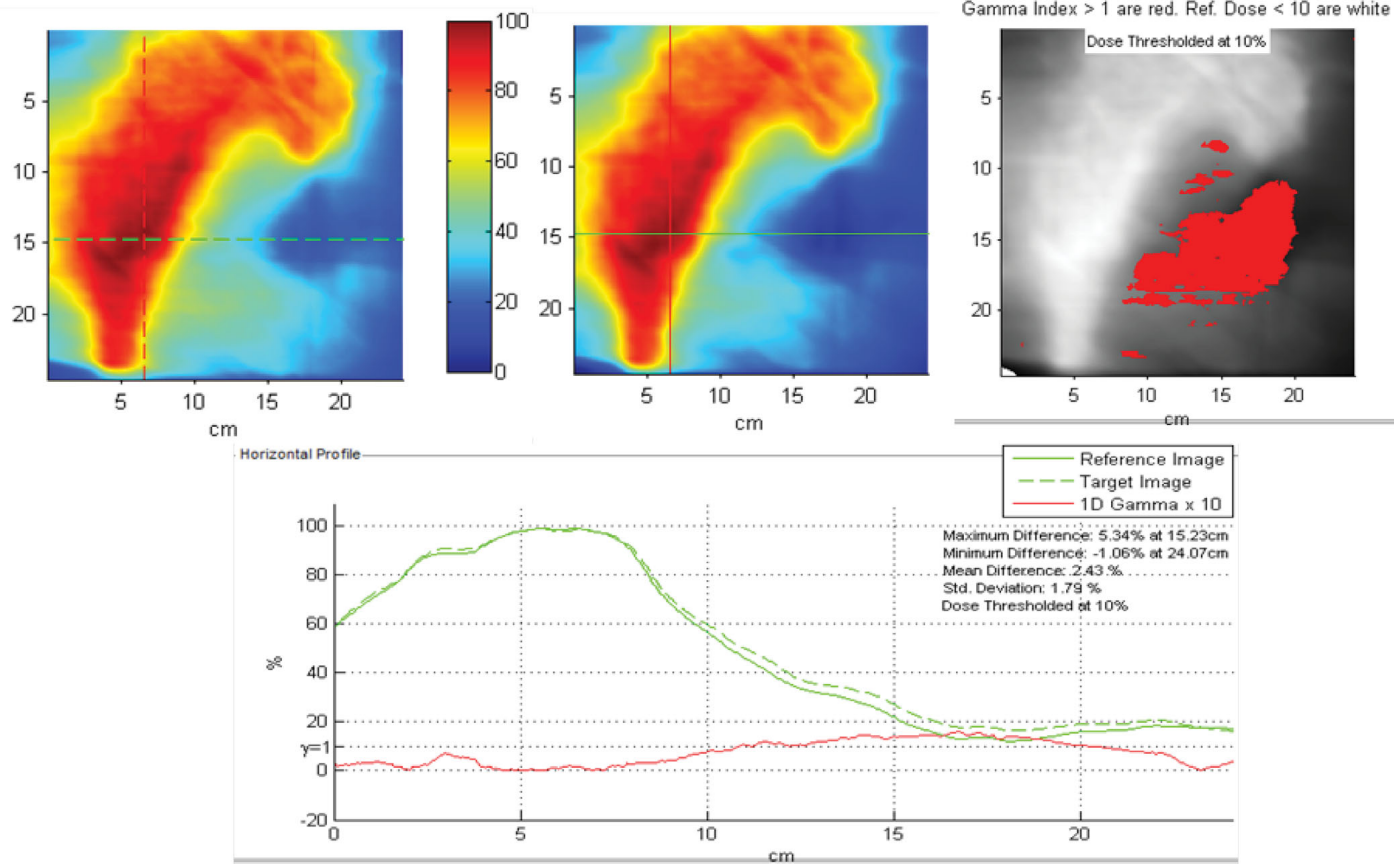
Example film result for an extended treatment field breast VMAT plan exhibiting over‐response to low‐energy scatter. Five of six extended field VMAT breast plans were below 95% passing rate on film with majority of failing points in the 20–40% dose range.

**Fig. 2:**
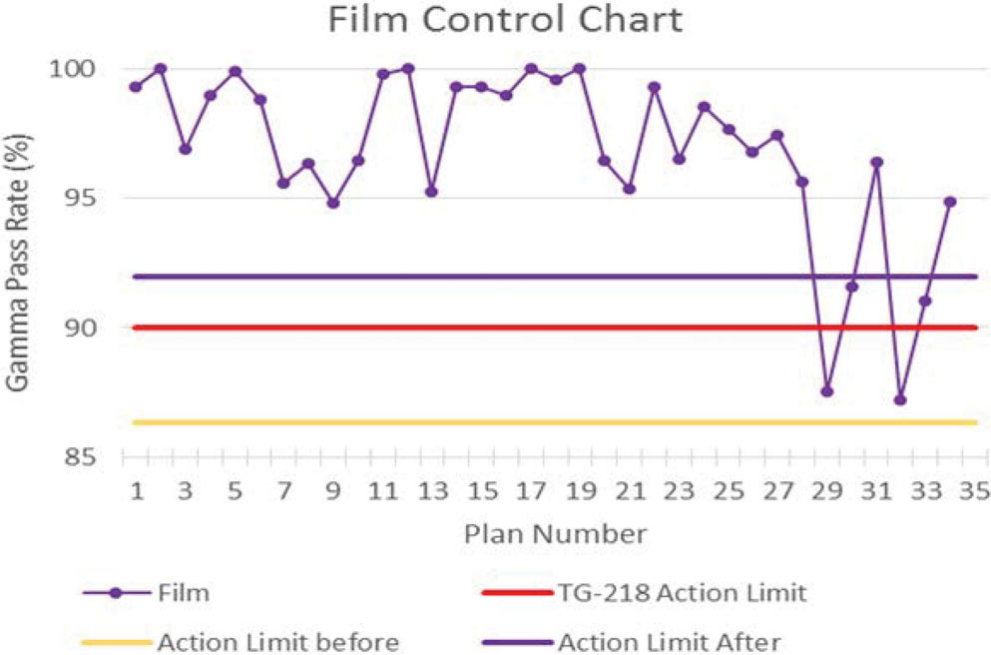
Control chart for EDR2 Film PSQA. The final six plans were with extended treatment field and where film over‐response is assumed. After removing these six from the action limit calculation, the limit increases to above TG‐218 recommendations.

Table 3 shows the mean IC percent difference and mean 3%/2 mm gamma passing rates for all measurement devices (TG‐119 plans included with ArcCHECK, Delta4, and PD). Values are shown for IMRT and VMAT separately as well as combined. The lower limit of the calculated tolerance and action limits are shown for all measurements (upper limit is set to 100%). 32 of 34 IC measurements were within 3% (recommended tolerance limit) and all were within 5% (recommended action limit) with an average difference of −0.6% and −0.9% for IMRT and VMAT respectively. The average passing rate for relative film measurements was 98.1% for IMRT and 96.3% for VMAT plans. Tolerance and action limits were calculated as 90.3% and 86.3% respectively. Five of the six extended field VMAT breast plans were below 95%, and the majority of the failing points were in the 20–40% dose range (Fig. 1). Since these plans utilize 10 arcs and large treatment fields, this may be attributed to EDR2 overresponse to low energy scatter.^25^ For one of these patient plans, we performed an IC measurement in the region failing on the film QA. Measured dose was 1.2% higher than expected relative to the prescription dose, which supports our hypothesis. Removing the extended treatment field films from tolerance and action limit calculations, the new limits were 93.3% and 92.0% respectively (Fig. 2).

**Table 3:** Ion chamber (IC) % difference and 3%/2 mm gamma passing rates (mean ± standard deviation) for all measurement devices for IMRT and VMAT plans.

	n	IC (%)	Film	n	ArcCHECK	Delta4	PD
IMRT	10	−0.6 ± 1.0	98.1 ± 1.9	15	99.1 ± 0.7	99.0 ± 1.1	99.8 ± 0.7
VMAT	24	−0.9 ± 1.6	96.3 ± 3.5	29	99.0 ± 0.9	99.3 ± 1.2	99.9 ± 0.4
ALL	34	−0.8 ± 1.4	96.8 ± 3.3	44	99.1 ± 0.9	99.2 ± 1.2	99.9 ± 0.5
Tolerance Limit			90.3		96.2	96.8	99.5
Action Limit			86.3		96.1	95.7	98.4

IC and film results do not include TG‐119 plans, while TG‐119 plans were measured with ArcCHECK, Delta4, and PD.

**Fig. 3:**
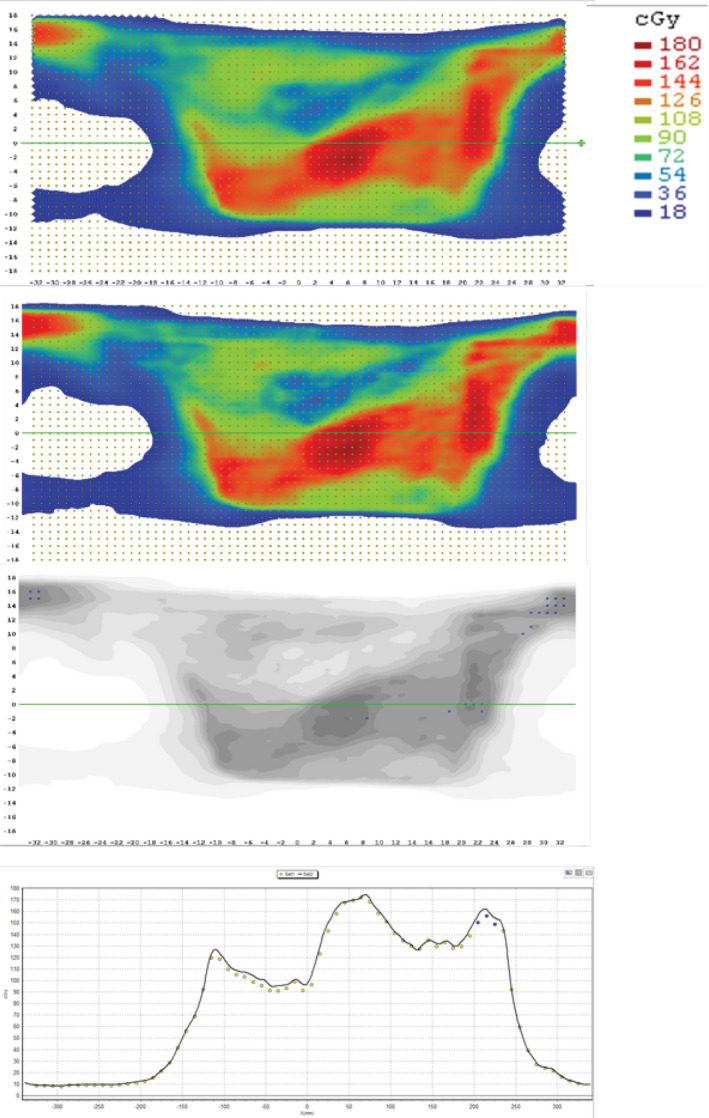
Extended treatment field delivered on ArcCHECK using the elongated treatment field measurement technique to capture the full dose distribution with automated couch shifts included in the delivery.

The average passing rates for ArcCHECK, Delta4, and PD were all above 99%. All plans had passing rates above 95%, including all six extended treatment field VMAT breast plans. A representative delivery of a breast VMAT plan using the extended treatment field technique is shown in Fig. 3. The action limits calculated were ArcCHECK: 96.1%, Delta4: 95.7%, and PD: 98.4%. These are well above the TG218 recommended action limit of 90%.

**Fig. 4:**
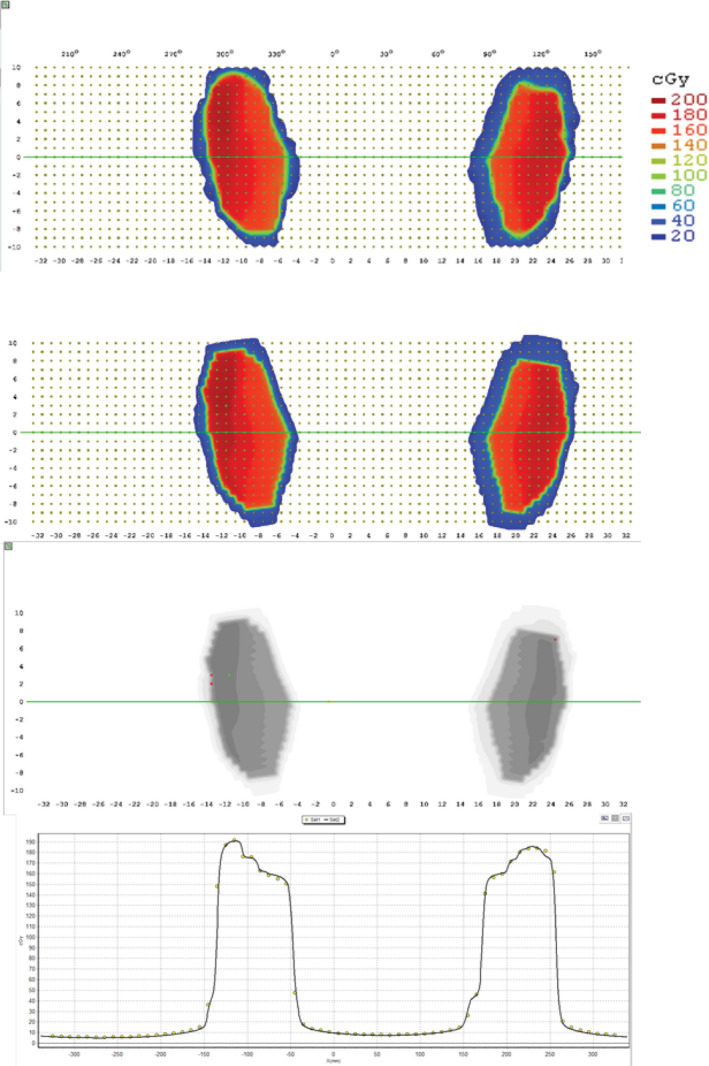
Tangent breast plan with dynamically flattened beam on ArcCHECK.

Table 4 shows the measured values for the 12 plans that utilize the DFB feature of the Halcyon. Figure 4 shows a representative tangent breast plan that utilized the DFB delivered on the ArcCHECK. Due to the limited number of plans and uniqueness of the fields (utilizes a fixed MLC sequence), these plans were not included in the tolerance and action limit calculations and treated separately. These results, however, seem to indicate that IC differences and gamma passing rates are comparable to the IMRT/VMAT plans.

**Table 4:** Mean ± SD point dose and true composite measured values for plans with DFB fields.

DFB plans	N	IC (%)	ArcCHECK	Delta4	PD
Breast	10	−1.5 ± 0.5	97.3 ± 1.7	99.4 ± 0.5	100 ± 0.0
AP/PA	1	−1.2	100	96.5	100
4 field box	1	−0.8	98.6	99.0	100

### 3.C Plan delivery stability

The gamma passing rate (Table 5) of two plans measured with ArcCHECK and PD had deviations of less than 1% with at least one month in‐between measurements.

**Table 5:** Gamma passing rates for two plans (one IMRT, one VMAT) measured with ArcCHECK (AC) and Portal Dosimetry (PD) with at least one month in between measurements.

	IMRT Plan AC	IMRT Plan PD	VMAT Plan AC	VMAT Plan PD
Measurement 1	99.7	100	98.9	100
Measurement 2	99.5	100	99.7	100
Measurement 3	98.9		99.8	

## 4 Discussion

This work validated the delivery of plans generated on a Halcyon 2.0 with preconfigured 6FFF beam model and independently movable stacks of MLC. Additionally, two new features, the extended treatment field and dynamically flattened beam were validated. TG 119 results showed good agreement with expected confidence limits and action limits. All 15 IMRT plans, 29 VMAT plans, and 12 DFB plans successfully surpassed the tolerance limit recommended by TG‐218 of >95% gamma passing rate with 3% dose difference (global normalization), 2 mm distance‐to‐agreement, and 10% dose threshold with the ArcCHECK, Delta4, and PD. Film results improved (average from 96.8% to 98.0%; tolerance 90.3% to 93.3%; action limit 86.3%–92.0%) when leaving out plans utilizing the extended treatment field.

The preconfigured beam model is expected to enable a shorter timeline from installation to clinical use. Although traditional commissioning procedures and fine‐tuning of beam models is no longer required, robust validation of the installed machine as it compares to the reference beam model remains vitally important. Of noted importance will be the validation of the delivery of clinical plans. The new double stack MLC with independently movable layers have a high degree of freedom to modulate the beam and create conformal treatment plans. We showed in this study that conventional approaches to PSQA are still appropriate with Halcyon 2.0. Routine MLC QA is recommended to check the MLC performance along with PSQA. Conventional PSQA approaches are known to be insensitive to some errors and have failures that do not relate to clinically relevant dose errors.^26^, ^27^ Therefore, validation of the delivery of clinical plans is only a portion of a robust validation of the installed machine. MPPG5.a provides guidelines for commissioning and quality assurance of treatment planning systems.^16^ This includes a full spectrum of validation tests that should be followed to commission the system.

The extended treatment field feature allows an 8 cm field size increase to 36 cm, only 4 cm smaller than traditional C‐arm LINACs in longitudinal direction. The field size increase helps the Halcyon unit to accommodate almost any size target traditionally treated on a C‐arm LINAC when using IMRT or VMAT. Thorough commissioning of the extended treatment field technique utilizing full field PSQA as done in this paper is recommended before clinical use. It is important to verify the full field composite dose utilizing the patient‐specific couch shifts. This requires knowledge of the procedure for measuring fields longer than the active area of the detector or measuring the plan twice at different locations to measure the full field. However, after commissioning the technique, routine QA of the couch motion and PSQA of the individual isocentre plans would be sufficient.

The DFB feature allows for simple treatment fields to be delivered with the Halcyon with similar dose distributions achieved with flattened beams. Many clinics may prefer these simple planning techniques, however, the dynamic MLCs of these fields need quality assurance. Performing patient‐specific QA on these plans appears to be an adequate QA procedure for these plans.

When measuring 3D breast DFB plans, adjustments to the application of angularly dependent correction factors may be needed. As the fields become more tangential to the ArcCHECK, the virtual inclinometer detects the gantry angle incorrectly as it is expecting entrance and exit doses on opposite sides of the phantom. Therefore, the gantry angle correction factor and angularly dependent heterogeneity correction factors would then be applied incorrectly resulting in erroneously low passing rates. The results of this paper have these correction factors turned off. Some of the plans tested were not as tangential and had good passing rates when the correction factors were included initially. These plans showed similar results after the correction factors were turned off. Adjustment from settings for IMRT/VMAT ArcCHECK measurements is not necessary in cases where the DFB fields are normally incident but, turning off these corrections factors will improve passing rates for tangentially oriented beams.

Results indicate that the calculated action limit might be tighter for Halcyon PSQA than the TG‐218 recommendations. Given the reference beam model all Halcyon treatment units are tuned to, PSQA results could be compared amongst Halcyon units. A future study comparing passing rates between institutions is ongoing to confirm interinstitutional consistency. If so, low resource clinics can quickly commission the unit and compare their PSQA results to these results to ensure the deliverability of high‐quality treatment plans. The reproducibility of the delivery with Halcyon was shown to be adequate. This is especially important for clinics to know that they can rely on consistent treatment delivery.

The main focus of this paper is to describe our experience in clinical validation of Halcyon clinical plans via measurement with multiple detector platforms, therefore, the plan quality comparison between Halcyon and C‐arm linac is outside the scope of this paper. There are many publications focusing on plan quality comparisons^7^, ^14^, ^28^, ^29^, ^30^, ^31^, ^32^ between Halcyon and C‐arm linacs which the reader is recommended to follow to get more information regarding plan quality evaluation. One limitation of this study is the number of plans delivered and number of sites. Although an attempt was made to cover the spectrum of potential disease sites and plan types, this dataset was biased toward breast VMAT plans with 6–10 partial arcs per plan (12 of 44 IMRT/VMAT). Due to the large field size and high modulation of these plans an argument could be made that these plans provide a good test of the robustness of the Halcyon plan delivery capability. This study also only measured one machine. A multi‐institutional study is underway and is needed to improve the knowledge of plan delivery reproducibility across machines. Lastly, a long‐term repeatability test is also needed to evaluate the consistency of plan delivery over a longer period of time than that evaluated in this study.

A wide variety of devices are utilized, but this is not intended to be an exhaustive list of options for performing PSQA nor recommend any one device over another. Comparisons among devices is difficult due to differences in detectors, geometry, analysis algorithms, etc. Yet, the intention is to provide a resource for those with the same Halcyon and PSQA device combinations as to what metrics and passing rates they reasonably should expect for a given combination.

## 5 Conclusion

Halcyon plan deliveries including new features available in v2.0 were verified with multiple measurement devices. Action limits for patient‐specific QA using the Halcyon treatment unit and a variety of measurement devices were calculated to be tighter than recommended in TG‐218. The manufacturing consistency between Halcyon units and pre‐defined beam model could allow for inter‐institutional comparison of PSQA values with a tighter action limit. A future study comparing inter‐institutional passing rates is needed to test this hypothesis. With favorable results, this could enable low resource clinics the ability to verify the deliverability of high‐quality treatment plans within multi‐institutional standards.

## Conflict of Interests

Dr. Cai reports grants from Varian Medical System, outside the submitted work. Dr. Kim reports grants and personal fees from Varian Medical, during the conduct of the study; grants and personal fees from Viewray, outside the submitted work. Dr. Hugo reports grants, personal fees, and other from Varian Medical Systems, during the conduct of the study; grants from Siemens Healthineers, grants from ViewRay, Inc., outside the submitted work. Dr. Mutic reports grants and other from Varian Medical Systems, Radialogica, TreatSafely, during the conduct of the study; grants, and other from Varian Medical Systems outside the submitted work.
